# Self-objectification, body uneasiness, and body investment in individuals undergoing body modification and plastic surgery: associations with depersonalization

**DOI:** 10.1007/s40519-025-01795-4

**Published:** 2025-11-21

**Authors:** Marzieh Abdoli, Fabio Carraturo, Dua Fatima Afzaal, Paolo Cotrufo, Stefania Cella

**Affiliations:** Observatory On Eating Disorders, Department of Psychology, University of Campania “Luigi Vanvitelli”, Caserta, Italy

**Keywords:** Body investment, Body modification, Body uneasiness, Depersonalization, Plastic surgery, Self-objectification

## Abstract

**Purpose:**

This study examined what is associated with self-objectification, body uneasiness, body investment, and depersonalization in adults who altered their bodies. We tested adults involved in body modification (BM) or plastic surgery (PS), and we compared them to controls to clarify these associations.

**Methods:**

We conducted an observational cross-sectional study of 235 adults (72.8% female) and grouped them into three categories: BM (*n* = 63), PS (*n* = 48), and controls (*n* = 124). Participants completed the Objectified Body Consciousness Scale, the Body Investment Scale, and the Body Uneasiness Test. We used analysis of covariance to compare groups while controlling for age and gender. We tested mediation by Body Investment Scale subscales and moderation by the number of BM and PS interventions in the relationship between self-objectification and depersonalization.

**Results:**

PS showed the highest self-objectification and body uneasiness, BM was intermediate, and controls were lowest. Body investment, particularly the body image subscale, mediated the association between self-objectification and depersonalization. The number of BM interventions strengthened the association between body investment and depersonalization, and PS tended to moderate the direct link between self-objectification and depersonalization.

**Conclusion:**

These findings suggest that body investment partly explains the connection between self-objectification and depersonalization, and that BM and PS relate differently to depersonalization in nonclinical adults. Given the cross-sectional design, we can only draw associative (not causal) conclusions.

Level of Evidence: III, observational cross-sectional study.

## Introduction

Identity, social expectations, and psychological conflict often center on the body [1, 2]. Over time, people have used body modification and cosmetic surgery to change their appearance in line with personal aims and local norms [3, 4]. Such changes can mark belonging, signal resistance, or express struggles with self-perception [5, 6]. Body modification (BM) and plastic surgery (PS) are not the same, yet they share a common psychological ground [7, 8]. BM includes tattoos and piercings, as well as scarification or implants. BM is cast as self-expression and, in some accounts, as vulnerability [9, 10]. It is also cast as empowerment and bodily ownership [11–13]. Other analyses associate BM with impulsivity, identity issues, and mental health concerns [14, 15]. Body investment shows different patterns across practices. Many piercings often come with lower body protection and higher self-monitoring, and symbolic tattoos can reflect identity goals [16, 17]. Plastic surgery comprises reconstructive and cosmetic procedures. Reconstructive work restores form and function. Cosmetic work is elective and aims at an appearance change. Here, we focus only on elective cosmetic procedures [18]. Internalized beauty ideals and body uneasiness often precede cosmetic surgery [19, 20]. Unlike some BM practices that challenge norms, cosmetic surgery usually seeks conformity and perceived enhancement [21, 22]. Studies associate elective procedures with higher self-objectification, more body uneasiness, and greater appearance-related distress [23, 24].

Self-objectification theory holds that a person can adopt an observer’s view of the body, which promotes self-surveillance and body shame. Media exposure, social comparison, and cultural messages sustain this process. Objectified body consciousness captures this stance and includes chronic monitoring of appearance, shame about the body, and beliefs about controlling appearance [25–28]. Repeated contact with objectifying content and appearance ideals is tied to stronger self-objectification [29, 30]. Higher objectified body consciousness co-occurs with body uneasiness, eating pathology, and interest in cosmetic surgery [31, 32]. Groups under closer scrutiny report more distress linked to body surveillance [33, 34]. These processes can fragment embodiment, so the body is treated as an object rather than part of an integrated self [35, 36]. Depersonalization is a dissociative state marked by detachment from one’s body and a sense of unreality [37–40]. High self-surveillance and body shame can weaken the sense of body ownership, which supports detachment. Persistent self-objectification may therefore contribute to depersonalization [41, 42]. Chronic self-monitoring and body uneasiness may also help explain why some people pursue modification practices to regain control [43, 44]. Those who are experiencing depersonalization commonly report a history of trauma, anxiety, along with body image-related disturbance, self-objectification, and compulsive appearance monitoring [45, 46]. People with higher depersonalization scores appear more likely to seek BM or cosmetic procedures for sensation or for change toward an idealized self [47, 48]. For some, modifying the body is an attempt to reconnect with lived bodily experience [49, 50]. Body investment is the degree of attachment, protection, and care directed to the body [51, 52]. Greater investment relates to valuing the body, health-protective actions, and resisting outside pressure [2, 53].

Lower body investment is tied to body uneasiness, self-neglect, and more modification behaviors [54, 55]. Motives and levels of body investment can differ between cosmetic surgery and BM. Elective surgery often aims at improvement or control of appearance. Some extreme BM practices co-occur with lower body protection and self-exploration, with wide variability across people and practices [56, 57]. Body uneasiness is distinct from body investment but tends to covary with it. Higher body uneasiness often appears with lower protective and caring attitudes and greater anxiety about the body [55, 58, 59]. High body uneasiness is common in disordered eating, body dysmorphic disorder, and compulsive modification tendencies [60, 61]. People who score high on body uneasiness more often pursue cosmetic surgery to reduce distress, yet procedures do not reliably reduce uneasiness over time [19, 23, 24, 62].

This study examines relations among objectified body consciousness, depersonalization, body investment, and body uneasiness in people who engage in BM and PS [63–65]. Three hypotheses guide the work. First, BM, PS, and control groups are compared on self-objectification, body investment, body uneasiness, and depersonalization, with the expectation of the highest self-objectification, body uneasiness, and depersonalization, and lower body protection, in PS, with BM in between, and controls lowest, H1. Second, body investment is expected to mediate the association between self-objectification and depersonalization, H2. Third, the number of body modifications and the number of elective surgeries are tested as moderators of the path from self-objectification to body investment and of the path from body investment to depersonalization, H3. Age and gender are included as covariates.

## Materials and methods

### Participants and procedure

A total of 235 adult participants, aged between 18 and 45, were recruited via convenience sampling. Participants were included if they met the age criterion and provided informed consent. Consent was obtained prior to the administration of the questionnaires. Participation was entirely voluntary, with no compensation. All procedures were in accordance with the ethical standards of the institutional research ethics committee and with the 1964 Declaration of Helsinki and its later amendments [66]. An initial pool of participants was identified among university students, who were then asked to involve their acquaintances in the study, particularly if they were known to have undergone body modification or plastic surgery procedures, by sharing a link to an online survey form. The study was presented as an inquiry into body image, body-related practices, and psychological traits; additional information was provided on request after survey completion. Participants completed a battery of self-report questionnaires preceded by a sociodemographic schedule and an ad hoc set of items inquiring about the number and type of body modification and plastic surgery interventions. Questionnaires were administered in a fixed order (sociodemographic schedule, BUT, BIS, OBCS). The initial pool of participants was constituted by psychology students enrolled in a course held by one of the authors. This author, however, was not involved directly in the data collection procedures. Participants who engaged in extreme body modification (scarification, ocular tattoos, implants, etc.) or who reported having > 4 piercings were included in the body modification group (BM; *N* = 63). Participants who reported having undergone plastic surgeries or intended to undergo one and did not engage in body modification practices (i.e., no extreme body modifications and ≤ 4 piercings) were included in the plastic surgery group (PS; *N* = 48). Participants who did not endorse either body modification or plastic surgery-related items were assigned to the control group (CN; *N* = 124). The > 4-piercing threshold was informed by prior work suggesting that having 3–5 piercings may co-occur with detrimental psychological traits and self-harm; we adopted a conservative cut-point (> 4) for operationalization, noting that tattoos/piercings do not necessarily imply maladjustment. Key sociodemographic characteristics by group are reported in Table 1. Age differed across groups (Kruskal–Wallis H(2, *N* = 235) = 17.483, *p* < 0.001), and gender distributions also differed (Pearson’s *χ*^2^(2) = 9.034, p = 0.011). The mean number of piercings was 0.581 (SD = 1.021) for controls, 11.683 (SD = 8.842) for BM, and 1.292 (SD = 1.473) for PS; the mean number of tattoos was 2.153 (SD = 4.191) for controls, 15.819 (SD = 18.738) for BM, and 1.292 (SD = 1.473) for PS.

**Table 1:** Sociodemographic characteristics of the sample

	Controls		Body modification		Plastic surgery		Full sample	
	*n*	%	*n*	%	*n*	%	*n*	%
Gender								
Female	83	66.9	45	71.4	43	89.6	171	72.8
Male	41	33.1	18	28.6	5	10.4	64	27.2
Age								
18–24	67	54.0	41	65.1	19	39.6	124	54.0
25–34	46	37.1	20	31.7	17	35.4	63	35.3
35–45	11	8.9	2	3.2	12	25.0	48	10.6
Sexual orientation								
Heterosexual	108	87.1	29	46.0	39	81.3	176	74.9
Homosexual	6	4.8	9	14.3	2	4.2	17	7.2
Bisexual	10	8.1	25	39.7	7	14.6	42	17.9
Other	0	0.0	0	0.0	0	0.0	0	0.0
Relationship status								
Single	53	42.7	30	47.6	13	27.1	96	40.9
In a stable relationship	71	57.3	33	52.4	35	72.9	139	59.1
Educational level								
Elementary school	1	0.8	4	6.3	0	0.0	5	2.1
Junior high-school	8	6.5	17	27.0	9	18.8	34	14.5
High-school	62	50.0	33	52.4	26	54.2	121	51.5
University	53	42.7	9	14.3	13	27.1	75	31.9
Citizenship								
Italian	123	99.2	60	95.2	48	100	231	98.3
Other	1	0.8	3	4.8	0	0.0	4	1.7
Reported self-harming behaviors								
Yes	10	8.1	23	36.5	3	6.3	36	15.3
No	114	91.9	23	63.5	3	93.8	199	84.7
Reported drug use								
Yes	10	8.1	16	25.4	3	6.3	29	12.3
No	114	91.9	47	74.6	45	93.8	206	87.7

The most frequent interventions carried out by participants in the plastic surgery group were: filler/botox (16.7%), mammoplasty (14.6%), rhinoplasty (14.6%), and liposuction (8.3%). Additional interventions undergone by one participant each were mastopexy, abdominoplasty, hair transplant, gastric bypass, and gynecomastia surgery. Two participants preferred not to disclose this information. The desired interventions instead included: mammoplasty (33.3%), rhinoplasty (25%), liposuction, and filler/botox (14.6% each), abdominoplasty (8.3%), and mastopexy (2.1%).

### Instruments

#### Sociodemographic schedule

The sociodemographic schedule addressed: gender, sex, sexual orientation, age, level of education, relationship status, citizenship, and also screened for substance abuse and self-harm with two dichotomous items. Additionally, the number of body modifications, plastic surgeries, piercings, and tattoos was inquired about.

#### Body uneasiness test

The Body Uneasiness Test (BUT; [59] is a 34-item assessment tool used to measure the discomfort related to bodily sensations, concerns related to body image, shape and appearance, and feelings of alienation from one’s body through five distinct factors of weight phobia (fear of becoming fat and weight-related anxieties), body image concerns (concerns related to appearance and body image), avoidance (behaviors and coping strategies to avoid distressing thoughts related to body image), compulsive self-monitoring (excessive monitoring of body appearance, weight, shape, and size of the body), and depersonalization (feeling of alienation and detachment from the body). It is a 6-point scale ranging from 0 (Not at All) to 5 (Always). Subscale and total scores are given by the average of the corresponding items. The subscales have been demonstrated to have good internal consistency with α ranging from 0.79 to 0.90, and factorial analysis supports a five-factor solution [59]. The original version of this instrument is in Italian [67]. The depersonalization subscale of the BUT was chosen for the moderated mediation analysis in this particular study for its emphasis on the corporeal aspects of self-dissociation.

#### Body Investment Scale

The Body Investment Scale (BIS; [52]. is designed to assess an individual's emotional investment in their body. BIS is a 24-item scale consisting of four subscales: positive body image (e.g., “I am satisfied with my body”), comfort with physical touch (e.g., “I enjoy physical contact with others”), body protection (e.g., “I am not afraid to engage in dangerous activities”), and body care (e.g., “I like to pamper my body”). Each subscale consists of 6 items measured on a 5-point Likert scale ranging from 1 (strongly disagree) to 5 (strongly agree). Scores for each subscale are calculated by averaging item responses, and higher scores indicate a more positive body investment. This scale has shown appropriate internal consistency levels (α between 0.75 and 0.92; [52]. An Italian translation of this instrument, which has confirmed the original factor structure, was used [68, 69].

#### Objectified Body Consciousness Scale

The Objectified Body Consciousness Scale (OBCS; [70] is a 24-item self-report measure designed to assess the degree to which a person views their body as an object that can be changed according to societal expectations. OBCS has three subscales: surveillance, viewing the body as an outsider; body shame, feelings of shame when the body does not conform to accepted standards; and appearance-control beliefs, believing that the appearance of the body is not fixed and can be changed by working on it. Each subscale has 8 items measured on a scale of 7 point Likert scale, with scores ranging from 1 (strongly disagree) to 7 (strongly agree). Scores are calculated for each subscale and for the overall scale by averaging the scores of each item. Higher scores indicate higher levels of each construct. This instrument has demonstrated a three-factor structure with good internal consistency (α between 0.78 and 0.82). The Italian version of the instrument has been validated and has replicated the original factor structure with good internal consistency of the three subscales (α between 0.78 and 0.92; [71].

### Data analysis

Descriptive statistics were obtained for all sociodemographic and psychometric variables. The Kolmogorov–Smirnov test was used to check if continuous variables were normally distributed to inform subsequent analyses [72]. One-way ANOVA and contingency tables were used to detect differences between groups in age and gender frequency, respectively [73, 74]. To account for significant differences in age and gender, further comparisons between groups were carried out through an ANCOVA in order to control for these two demographic variables [74]. Significance levels were adjusted for multiple post hoc comparisons using the Holm–Bonferroni correction. For non-normally distributed variables, Quade’s ANCOVA was selected as a nonparametric alternative [75, 76]. Post hoc contrasts are reported as adjusted means with Holm–Bonferroni-corrected two-tailed *p* values at *α* = 0.05.

This cross-sectional study conducted a regression analysis with objectified body consciousness as a predictor, body investment subdimensions (BIS subscales) as mediators, and the BUT Depersonalization subscale as the criterion (outcome). The number of body modification interventions and the number of plastic surgeries were included in the model as moderators of the effect of objectification and of body investment on depersonalization. Specifically, we tested a moderated-mediation model (PROCESS macro for SPSS, template 17) in which (a) the path(s) from each BIS mediator to depersonalization and (b) the direct path from objectification to depersonalization were moderated by the counts of body modification (BM) and plastic surgery (PS) interventions. All continuous predictors were mean-centered prior to creating interaction terms. Age and gender were controlled as covariates. For the regression model, a bootstrapping procedure with 5000 resamples was applied using the PROCESS macro for SPSS, template 17 [77], with 95% bias-corrected confidence intervals. All remaining analyses were run via the default functions of SPSS (v. 29.0.1.0).

## Results

As displayed in Table 2, several significant differences emerged on the assessed psychometric variables between the three groups while controlling for age and gender.

**Table 2:** ANCOVA comparison between groups

Variable name	Control group		Body modification		Plastic surgery		ANCOVA	
	M	SD	M	SD	M	SD	*F(df1, df2)*	*p*
OBCS surveillance*	4.102^a^	0.965	4.435^a^	0.999	5.083^b^	0.934	16.525_2, 230_	** < 0.001**
OBCS shame*	3.263^a^	1.194	3.661^a^	1.376	4.555^b^	1.317	14.262_2, 228_	** < 0.001**
OBCS control beliefs*	4.857^a^	0.792	4.515^b^	0.742	4.258^b^	0.914	7.867_2, 228_	** < 0.001**
OBCS total score*	4.074^a^	0.598	4.233^a^	0.723	4.632^b^	0.559	13.074_2, 230_	** < 0.001**
BIS body image	3.817^a^	0.833	3.331^b^	1.084	2.677^c^	1.152	16.401_2, 232_	** < 0.001**
BIS body touch*	3.273	0.899	3.069	0.893	3.306	0.892	1.144_2, 230_	0.320
BIS body care	3.723	0.654	3.792	0.718	4.000	0.541	2.349_2, 232_	0.098
BIS body protection	4.106^a^	0.690	3.393^b^	0.809	3.965^a^	0.818	14.064_2, 232_	** < 0.001**
BIS total score	89.52^a^	11.80	81.74^b^	14.26	83.69^b^	14.32	6.527_2, 232_	**0.002**
BUT weight phobia	2.559^a^	1.231	3.439^b^	1.400	4.383^c^	1.370	29.123_2, 232_	** < 0.001**
BUT body image concerns	2.313^a^	1.094	3.007^b^	1.402	3.849^c^	1.491	18.226_2, 232_	** < 0.001**
BUT avoidance	1.518^a^	0.827	1.984^b^	1.190	2.493^b^	1.374	11.409_2, 232_	** < 0.001**
BUT compulsive self-monitoring	2.090^a^	0.988	2.756^b^	1.191	3.281^c^	1.249	20.665_2, 232_	** < 0.001**
BUT depersonalization	1.465^a^	0.854	2.126^b^	1.253	2.779^c^	1.611	16.688_2, 232_	** < 0.001**
BUT total score	4.074^a^	0.598	4.233^b^	0.723	4.632^c^	0.559	24.611_2, 232_	** < 0.001**

Variables marked with an asterisk were not normally distributed (as indicated by a significant Kolmogorov–Smirnov test), so Quade nonparametric ANCOVA was performed. For all other variables, a parametric ANCOVA was chosen. Letters a, b, and c next to mean values indicate significant post-hoc differences between groups with α = 0.05 adjusted for multiple comparisons using the Holm–Bonferroni method, i.e., mean values that share the same letter do not significantly differ from one another

With the exception of Body Care and Body Touch, controls showed consistently lower self-objectification and body image disturbances compared to participants who engaged in body modification and plastic surgery. Although not all post hoc comparisons yielded significant differences in each pairing, a general trend emerged: the plastic surgery group displayed the highest self-objectification, the lowest body investment, and the highest body image disturbances, followed by the body modification and finally by the control group. The only variable that did not conform to this pattern was the control beliefs subscale of the OBCS, for which the control group reported the highest values.

**Fig. 1:**
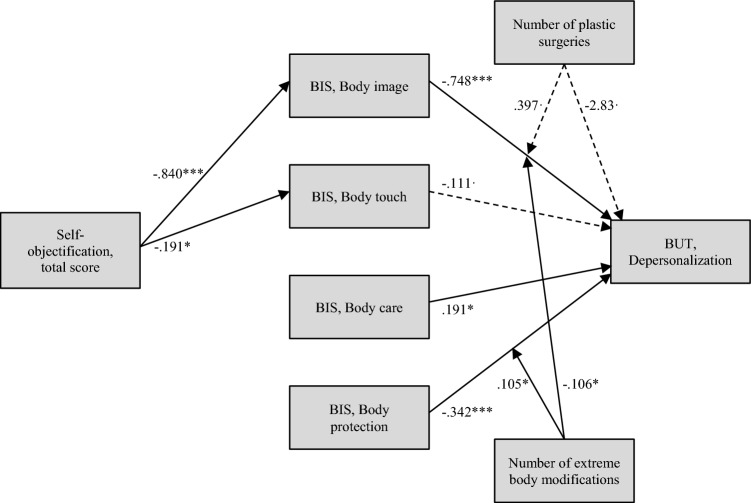
Mediation analysis. Continuous lines depict significant effects at alpha < .05, dotted lines depict quasi-significant effects at alpha < 0.10. ***p < 0.001, ** = *p* < 0.01, * = *p* < 0.05,  = *p* < 0.10

Mediation analysis (Fig. 1) revealed a significant indirect effect of the total objectification score on depersonalization through the body image subscale: *b* = 0.629, LLCI = 0.408, ULCI = 0.901. The total objectification score did not have a direct unconditional effect on depersonalization, indicating that the mediation through the body image subscale was total: *b* = 0.068, *p* = 0.491, LLCI = −0.127, ULCI = 0.263. With the exception of body touch, which had a near significant effect, all subscales of the BIS significantly predicted depersonalization scores: body image had a negative direct effect, *b*** = **−0.748, *p* < 0.001, LLCI = −0.885, ULCI = −0.611; body care had a positive direct effect, *b*** = **0.195, *p* = 0.032, LLCI = 0.018, ULCI = 0.373; body protection had a negative direct effect, *b*** = **−0.342, *p* < 0.001, LLCI = −0.489, ULCI = −0.194. Moreover, the number of extreme body modifications moderated some of these relationships, enhancing the negative effect of the body image subscale (*b*** = **−0.105, *p* = 0.025, LLCI = −0.198, ULCI = -0.013) and reverting the negative effect of body protection (*b*** = **0.106, *p* = 0.015, LLCI = 0.020, ULCI = 0.191). As an illustration, for zero, one, and three body modifications, the effect of the body image subscale on depersonalization while controlling for plastic surgery is, respectively, −0.752, −0.804, and −0.986. For all these conditional effects, *p-*values are below 0.001. The effect of body protection is −0.334 for zero body modifications (*p* < 0.001), −0.286 for one body modification (*p* < 0.001), and −0.104 for three body modifications; however, for this last value, there is a loss of statistical significance (*p* 0.341). With regard to moderated effects, the unmediated relationship between the total objectification score and depersonalization was only significant when at least one plastic surgery had been done, but not when plastic surgery was absent, and the interaction between plastic surgery and objectification approached significance: *b*** = **0.396, *p* = 0.053, LLCI = −0.005, ULCI = 0.798. The relationship between the number of plastic surgery interventions and depersonalization did not reach statistical significance: *b*** = **−2.823, *p* = *0.0*72, LLCI = −5.915, ULCI = 0.258. Overall, the model explained 67% of the variance of depersonalization (R^2^ = 0.672, *F(19, 214)* = *23.059, p* < *0.001*).

## Discussion

The present findings of this study show clear differences across the three groups. Plastic surgery patients had the highest levels of self-objectification, body uneasiness, and depersonalization; individuals with body modifications had moderate levels, and the control group had the lowest. This pattern suggests that elective cosmetic surgery is associated with higher self-objectification and depersonalization, whereas body modification shows intermediate values. We avoid causal language and over-interpretation; instead, we note that our group pattern aligns with scholarship framing cosmetic procedures as more conformity-oriented and objectification-consistent than many BM practices [5, 6, 78, 79]. Notably, controls scored highest on OBCS control beliefs and on BIS body protection and BIS total, indicating comparatively stronger perceived appearance-control beliefs and protective attitudes in this group. The lack of significant differences on the care and touch subscales of the BIS may be due to potential differences between subgroups being qualitative rather than quantitative (like seeking sensual contact with others to attain intimacy rather than to instrumentally induce feelings of pleasure), which may go undetected with the use of self-report, standardized measures. Our mediation analysis showed that body investment plays a complex role in depersonalization. Higher scores on the BIS body image and body protection subscales were associated with lower depersonalization, whereas higher scores on the body care subscale were associated with higher depersonalization. Taken together, valuing and protecting one’s body were associated with lower detachment experiences, whereas a stronger emphasis on appearance-focused care was associated with higher depersonalization. These findings imply that valuing and safeguarding one’s body may buffer against feelings of detachment, while an appearance focus may intensify them. The interplay between body investment and depersonalization provides further insight. Moderation by BM count was directional: as the number of extreme body modifications increased, the negative association between BIS body image and depersonalization became stronger (from approximately − 0.75 at 0 BM to ~  − 0.99 at 3 BM), whereas the negative association of BIS body protection weakened and could become non-significant at higher BM counts. Plastic surgery, while also involving bodily interventions, differs in its motivations. Those who undergo PS aim not only to assert control over their appearance, but also to enhance desirability and conform to societal ideals. Unlike in the control and PS groups, where body protection may signal a more conventional sense of bodily care, in the BM group, it may indicate a shift in how bodily boundaries are perceived. This is consistent with the idea that body modification extends beyond aesthetics, and for some individuals may function as an unsuccessful attempt to reclaim control over the body and actively reshape self-perception. Prior research suggests that individuals with elevated depersonalization often pursue bodily alterations to restore a sense of reality and counter the numbing effects of self-objectification. This is consistent with accounts that view body modification as a transitional step from self-alienation to the body’s materiality, a theme grounded in theories of embodiment and subjectivity [80–83].

The PS × objectification interaction was small (b≈0.40) with a wide confidence interval that narrowly crossed zero; we therefore treat this pattern as tentative and in need of replication. This distinction underlines how body modification and plastic surgery diverge: the former may challenge dominant aesthetic norms, while the latter often reinforces external validation, which may set up a cycle in which dissatisfaction is displaced rather than resolved [23, 51, 56, 84]. The moderating effect of how many procedures have been undertaken adds nuance. Higher counts of extreme modifications are linked with a tighter coupling between body image distress and depersonalization. This implies that acts meant to reclaim the body can also intensify attention to bodily states. Prior work likewise points to repeated modification as a marker of continuing struggles with embodiment rather than a full resolution of dissociation [85, 86]. Conversely, for plastic surgery, the link between self-objectification and depersonalization reached significance only when at least one procedure had been undertaken, and the effect under that condition was small, consistent with the view that such procedures chiefly serve conformity to external beauty ideals rather than acts of bodily self-ownership [1, 9, 19, 87]. The results support the view that body modification and plastic surgery engage distinct psychological registers. At its most extreme, body modification signals a bid to repossess the body by intentional interventions, treating it as a possessed object and aiming at “making the self real” through physical experience. The act of change functions as an inscription and an assertion of agency to offset detachment. Plastic surgery, by comparison, is more closely tied to cultural narratives of perfection and control. These findings suggest that while surgery may momentarily alleviate body-related distress, it does not fundamentally alter the internalized gaze through which individuals evaluate themselves. Instead, the cycle of dissatisfaction may persist, reinforcing a fragmented sense of embodiment where the self is continuously measured against an unattainable ideal [50, 88–90]. Findings build on objectification theory by showing that body investment plays an important role in the link between self-objectification and depersonalization. The results suggest that strengthening positive body investment may help reduce feelings of detachment. In practice, interventions that lower self-objectification and promote body investment could help ease depersonalization and body uneasiness [33].

Lastly, the findings underscore the need for interventions that address self-objectification and body image disturbances through a lens that prioritizes embodiment. Common behavioral approaches may be insufficient in cases where depersonalization is a core component of body-related distress. Rather than standard symptom-focused approaches, therapies that cultivate embodiment, integrating a coherent sense of the bodily self and attending to the psychological motives behind modification, may better help align self-perception with lived physical experience [91, 92]. Taken together, our findings describe how people relate to their bodies under self-objectification, body investment, and depersonalization. Modification can be a bid to reclaim the body, but its benefits are partial and depend on the form it takes. It may bolster ownership without fully resolving dissociative experiences and can be tinged with hostility toward the body, offering relief that stops short of a lasting solution to psychological distress. Plastic surgery, on the other hand, seems to depend on and reinforce societal beauty standards. Furthermore, these interventions may be partial attempts to deal with embodiment, yet they can act in varied ways. This study shows that interventions aimed at the body are not just matters of aesthetics but are deeply anchored responses to concerns of selfhood, identity, and psychological conflict.

### Strengths and limitations

The inclusion of three comparison groups (body modification, plastic surgery, controls), use of validated Italian versions of the OBCS, BIS, and BUT, and adjustment for age and gender using both parametric and nonparametric ANCOVA, plus a moderated mediation framework.

The cross-sectional, convenience design; self-report measures with fixed order; web-based recruitment with potential selection bias; female-skewed sample; absence of clinical interviews, motivation measures, and comorbidity assessments; and a focus on depersonalization without broader dissociative symptoms.

## What is already known about this subject?

Self-objectification and body uneasiness are linked to interest in aesthetic procedures, and depersonalization reflects detachment from the bodily self. Body investment (valuing, caring for, and protecting the body) is thought to buffer body-related distress, but its role in the self-objectification–depersonalization link is unclear, particularly when comparing body modification and cosmetic surgery.

## What this study adds

In nonclinical adults, cosmetic-surgery participants showed the highest self-objectification, body uneasiness, and depersonalization; body-modification participants were intermediate; and controls were lowest. Body investment, especially the body image facet, mediated the association between self-objectification and depersonalization, and the number of extreme body modifications strengthened this link. These findings identify body investment as a tractable target for interventions addressing depersonalization among people seeking appearance-altering practices.

## Limitations and future directions

This study has several limitations that should be considered when interpreting the findings. Because the data were collected at a single point in time, it is not possible to know whether self-objectification leads to depersonalization or whether individuals who already feel detached from their bodies become more self-objectified. Accordingly, whether surgical or modification practices reduce, exacerbate, or temporarily relieve depersonalization cannot be determined without longitudinal designs [93]. Longitudinal research is needed to clarify this direction. All variables were measured with self-report questionnaires, which may be influenced by social desirability or inaccurate recall, and by common-method variance and fixed questionnaire order [94]. Using clinical interviews, behavioral observations, or implicit measures in future studies could provide more objective assessments of self-objectification, body investment, and depersonalization. The sample was recruited online. Although it included a range of ages and both genders, it may not represent people from other cultures or with different demographic backgrounds and may be affected by selection bias typical of nonprobability web surveys [95]. Future studies should therefore include participants from diverse cultural groups and from clinical populations, such as individuals with body dysmorphic disorder or trauma histories, to improve generalizability. Male participants were included in all analyses to preserve statistical power, even though women represented the majority in all subsamples. Gender was controlled as a covariate in the statistical analyses. Still, because body image and self-objectification concerns are more common among women, this remains a clear limitation. Future work should examine whether the mechanisms discussed here operate differently across genders. The study did not consider participants’ psychological motivations for undergoing body modification or cosmetic surgery.

Understanding why people choose these interventions could provide insights into the processes that link self-objectification and body investment. Relatedly, comorbid mental health conditions, such as depression or anxiety, were not measured, even though they may affect body image and dissociative experiences. Including these variables in future studies would help clarify their role. Details about the number, type, or location of tattoos, piercings, and surgeries were not collected. These factors may relate differently to self-objectification, body investment, and depersonalization, so they should be recorded in future research. The study focused only on depersonalization and did not include other dissociative symptoms, such as derealization or identity disturbance [96]. Examining a broader range of dissociative experiences would allow for a more complete understanding of how self-objectification affects mental health. Finally, the study did not analyze data separately for men and women, even though gender differences in body image and self-objectification are well-documented. Future work should examine men and women separately to identify gender differences. Studying these questions over time and in different cultures may help develop interventions that promote healthy body investment and lessen self-objectification and depersonalization.

## Conclusion

In conclusion, participants who had cosmetic surgery reported the highest levels of self‑objectification, body uneasiness, and depersonalization; those with body modifications reported intermediate levels, and controls reported the lowest levels. Body investment partly mediated the association between self‑objectification and depersonalization, and the number of body modifications or surgeries moderated these associations. These findings suggest that self‑objectification and body investment are key factors in understanding depersonalization and body uneasiness among people who alter their bodies. Because this study was cross‑sectional and relied on self‑report measures, we cannot draw causal conclusions. Future research should examine these relationships over time and explore why different types of body alterations are associated with varying psychological outcomes.

## Data Availability

The datasets for this study are available from the authors upon reasonable request.

## References

[1] Pitts V (2003) In the flesh: the cultural politics of body modification. Palgrave Macmillan, London

[2] Swami V, Pietschnig J, Bertl B et al (2012) Personality differences between tattooed and non-tattooed individuals. Psychol Rep 111:97–106. 10.2466/09.07.21.PR0.111.4.97-106 23045851 10.2466/09.07.21.PR0.111.4.97-106

[3] Dion K, Berscheid E, Walster E (1972) What is beautiful is good. J Pers Soc Psychol 24:285–290. 10.1037/h0033731 4655540 10.1037/h0033731

[4] Sweetman P (1999) Anchoring the (Postmodern) self? Body modification, fashion and identity. Body Soc 5:51–76. 10.1177/1357034X99005002004

[5] Atkinson M (2004) Tattooing and civilizing processes: body modification as self-control. Can Rev Sociol Anthropol 41:125–146. 10.1111/j.1755-618x.2004.tb02173.x 15290832 10.1111/j.1755-618x.2004.tb02173.x

[6] Featherstone M (2010) Body, image and affect in consumer culture. Body Soc 16:193–221. 10.1177/1357034X09354357

[7] Blascovich J, Mendes WB (2010) Social psychophysiology and embodiment. In: Fiske ST, Gilbert DT, Lindzey G (eds) Handbook of social psychology. John Wiley & Sons Ltd, Hoboken

[8] Gyllensten AL, Skär L, Miller M, Gard G (2010) Embodied identity–a deeper understanding of body awareness. Physiother Theory Pract 26:439–446. 10.3109/09593980903422956 20649495 10.3109/09593980903422956

[9] Stirn A, Hinz A (2008) Tattoos, body piercings, and self-injury: is there a connection? Investigations on a core group of participants practicing body modification. Psychother Res 18:326–333. 10.1080/10503300701506938 18815984 10.1080/10503300701506938

[10] Wohlrab S, Stahl J, Kappeler PM (2007) Modifying the body: motivations for getting tattooed and pierced. Body Image 4:87–95. 10.1016/j.bodyim.2006.12.001 18089255 10.1016/j.bodyim.2006.12.001

[11] Jabłońska K, Mirucka B (2023) Mental body representations of women with tattoos in emerging adulthood — a cluster analysis. Arch Womens Ment Health 26:473–483. 10.1007/s00737-023-01326-z 37261495 10.1007/s00737-023-01326-z PMC10333373

[12] Kosut M (2000) Tattoo narratives: the intersection of the body, self-identity and society. Vis Sociol 15:79–100. 10.1080/14725860008583817

[13] Mun JM, Janigo KA, Johnson KKP (2012) Tattoo and the self. Cloth Text Res J 30:134–148. 10.1177/0887302X12449200

[14] Claes L, Vandereycken W, Vertommen H (2001) Self-injurious behaviors in eating-disordered patients. Eat Behav 2:263–272. 10.1016/s1471-0153(01)00033-2 15001035 10.1016/s1471-0153(01)00033-2

[15] Stirn A, Hinz A, Brähler E (2006) Prevalence of tattooing and body piercing in Germany and perception of health, mental disorders, and sensation seeking among tattooed and body-pierced individuals. J Psychosom Res 60:531–534. 10.1016/j.jpsychores.2005.09.002 16650594 10.1016/j.jpsychores.2005.09.002

[16] Lammek MJ (2022) The body as a reflection of relations with others. Curr Issues Pers Psychol 10:184–189. 10.5114/cipp.2022.112928 10.5114/cipp.2022.112928 PMC10535630 38013823

[17] Tiggemann M, Hopkins LA (2011) Tattoos and piercings: bodily expressions of uniqueness? Body Image 8:245–250. 10.1016/j.bodyim.2011.03.007 21561820 10.1016/j.bodyim.2011.03.007

[18] McInnes CW, Courtemanche DJ, Verchere CG et al (2012) Reconstructive or cosmetic plastic surgery? Factors influencing the type of practice established by Canadian plastic surgeons. Can J Plast Surg 20:163–168. 10.1177/229255031202000312 23997582 10.1177/229255031202000312 PMC3433812

[19] Crerand CE, Franklin ME, Sarwer DB (2006) Body dysmorphic disorder and cosmetic surgery. Plast Reconstr Surg 118:167e–180e. 10.1097/01.prs.0000242500.28431.24 17102719 10.1097/01.prs.0000242500.28431.24

[20] Sarwer DB, Wadden TA, Pertschuk MJ, Whitaker LA (1998) Body image dissatisfaction and body dysmorphic disorder in 100 cosmetic surgery patients. Plast Reconstr Surg 101:1644–1649. 10.1097/00006534-199805000-00035 9583501 10.1097/00006534-199805000-00035

[21] Frederick DA, Lever J, Peplau LA (2007) Interest in cosmetic surgery and body image: views of men and women across the lifespan. Plast Reconstr Surg 120:1407–1415. 10.1097/01.prs.0000279375.26157.64 17898621 10.1097/01.prs.0000279375.26157.64

[22] Swami V, Arteche A, Chamorro-Premuzic T et al (2008) Looking good: factors affecting the likelihood of having cosmetic surgery. Eur J Plast Surg 30:211–218. 10.1007/s00238-007-0185-z

[23] Javo IM, Sørlie T (2009) Psychosocial predictors of an interest in cosmetic surgery among young Norwegian women: a population-based study. Plast Reconstr Surg 124:2142–2148. 10.1097/PRS.0b013e3181bcf290 19952672 10.1097/PRS.0b013e3181bcf290

[24] Sarwer DB, Crerand CE (2004) Body image and cosmetic medical treatments. Body Image 1:99–111. 10.1016/S1740-1445(03)00003-2 18089144 10.1016/S1740-1445(03)00003-2

[25] Calogero RM (2012) Objectification theory, self-objectification, and body image. In: Cash T (ed) Encyclopedia of body image and human appearance. Elsevier Academic Press, San Diego, pp 574–580

[26] Fredrickson BL (2001) The role of positive emotions in positive psychology. Am Psychol 56:218–226 11315248 10.1037//0003-066x.56.3.218 PMC3122271

[27] Moradi B, Huang Y-P (2008) Objectification theory and psychology of women: a decade of advances and future directions. Psychol Women Q 32:377–398. 10.1111/j.1471-6402.2008.00452.x

[28] Tiggemann M, Lynch JE (2001) Body image across the life span in adult women: the role of self-objectification. Dev Psychol 37:243–253. 10.1037/0012-1649.37.2.243 11269392 10.1037/0012-1649.37.2.243

[29] Grabe S, Ward LM, Hyde JS (2008) The role of the media in body image concerns among women: a meta-analysis of experimental and correlational studies. Psychol Bull 134:460–476. 10.1037/0033-2909.134.3.460 18444705 10.1037/0033-2909.134.3.460

[30] Vandenbosch L, Eggermont S (2012) Understanding sexual objectification: a comprehensive approach toward media exposure and girls’ internalization of beauty ideals, self-objectification, and body surveillance. J Commun 62:869–887. 10.1111/j.1460-2466.2012.01667.x

[31] Calogero RM, Jost JT (2011) Self-subjugation among women: exposure to sexist ideology, self-objectification, and the protective function of the need to avoid closure. J Pers Soc Psychol 100:211–228. 10.1037/a0021864 21186932 10.1037/a0021864

[32] Fardouly J, Diedrichs PC, Vartanian LR, Halliwell E (2015) Social comparisons on social media: the impact of Facebook on young women’s body image concerns and mood. Body Image 13:38–45. 10.1016/j.bodyim.2014.12.002 25615425 10.1016/j.bodyim.2014.12.002

[33] Rollero C, Gattino S, De Piccoli N, Fedi A (2018) Protective versus risk factors for self-objectification in different age and gender cohorts. Psihologija 51:17–30. 10.2298/PSI161222008R

[34] Tylka TL, Hill MS (2004) Objectification theory as it relates to disordered eating among college women. Sex Roles 51:719–730. 10.1007/s11199-004-0721-2

[35] Corno G, Paquette A, Monthuy-Blanc J et al (2022) The relationship between women’s negative body image and disordered eating behaviors during the COVID-19 pandemic: a cross-sectional study. Front Psychol 13:856933. 10.3389/fpsyg.2022.856933 35401386 10.3389/fpsyg.2022.856933 PMC8987766

[36] Tantleff-Dunn S, Barnes RD, Larose JG (2011) It’s not just a “woman thing:” the current state of normative discontent. Eat Disord 19:392–402. 10.1080/10640266.2011.609088 21932970 10.1080/10640266.2011.609088 PMC3760219

[37] Medford N (2012) Emotion and the unreal self: depersonalization disorder and de-affectualization. Emot Rev 4:139–144. 10.1177/1754073911430135

[38] Michal M, Wiltink J, Subic-Wrana C et al (2009) Prevalence, correlates, and predictors of depersonalization experiences in the German general population. J Nerv Ment Dis 197:499–506. 10.1097/NMD.0b013e3181aacd94 19597357 10.1097/NMD.0b013e3181aacd94

[39] Sierra M, Berrios GE (2001) The phenomenological stability of depersonalization: comparing the old with the new. J Nerv Ment Dis 189:629–636. 10.1097/00005053-200109000-00010 11580008 10.1097/00005053-200109000-00010

[40] Simeon D, Abugel J (2006) Feeling unreal: depersonalization disorder and the loss of the self. Oxford University Press, New York

[41] Levin KK, Gornish A, Quigley L (2022) Mindfulness and depersonalization: a nuanced relationship. Mindfulness (N Y) 13:1479–1489. 10.1007/s12671-022-01890-y 35492870 10.1007/s12671-022-01890-y PMC9043097

[42] Loughnan S, Haslam N, Murnane T et al (2010) Objectification leads to depersonalization: the denial of mind and moral concern to objectified others. Eur J Soc Psychol 40:709–717. 10.1002/ejsp.755

[43] Dakanalis A, Timko CA, Zanetti MA et al (2014) Attachment insecurities, maladaptive perfectionism, and eating disorder symptoms: a latent mediated and moderated structural equation modeling analysis across diagnostic groups. Psychiatry Res 215:176–184. 10.1016/j.psychres.2013.10.039 24295762 10.1016/j.psychres.2013.10.039

[44] Piran N (2016) Embodied possibilities and disruptions: the emergence of the experience of embodiment construct from qualitative studies with girls and women. Body Image 18:43–60. 10.1016/j.bodyim.2016.04.007 27236476 10.1016/j.bodyim.2016.04.007

[45] Gómez-Peresmitré G, Platas-Acevedo RS (2023) Depression disorders in Mexican adolescents: a predictive model. Children (Basel) 10:1264. 10.3390/children10071264 37508761 10.3390/children10071264 PMC10377976

[46] Yang J, Millman LSM, David AS, Hunter ECM (2023) The prevalence of depersonalization-derealization disorder: a systematic review. J Trauma Dissociation 24:8–41. 10.1080/15299732.2022.2079796 35699456 10.1080/15299732.2022.2079796

[47] Feingold A, Mazzella R (1998) Gender differences in body image are increasing. Psychol Sci 9:190–195. 10.1111/1467-9280.00036

[48] Merleau-Ponty M (1962) Phenomenology of perception. Routledge, London

[49] Ataria Y (2015) Sense of ownership and sense of agency during trauma. Phenom Cogn Sci 14:199–212. 10.1007/s11097-013-9334-y

[50] Dolezal L (2015) The body and shame: phenomenology, feminism, and the socially shaped body. Lexington Books, Lanham

[51] Cash TF, Pruzinsky T (2004) Body image: a handbook of theory, research, and clinical practice. Guilford Publications, New York

[52] Orbach I, Mikulincer M (1998) The body investment scale: construction and validation of a body experience scale. Psychol Assess 10:415–425. 10.1037/1040-3590.10.4.415

[53] Brown TA, Cash TF, Mikulka PJ (1990) Attitudinal body-image assessment: factor analysis of the body-self relations questionnaire. J Pers Assess 55:135–144. 10.1080/00223891.1990.9674053 2231236 10.1080/00223891.1990.9674053

[54] Jung J, Lee S-H (2006) Cross-cultural comparisons of appearance self-schema, body image, self-esteem, and dieting behavior between Korean and U.S. women. Fam Consum Sci Res J 34:350–365. 10.1177/1077727X06286419

[55] Noles SW, Cash TF, Winstead BA (1985) Body image, physical attractiveness, and depression. J Consult Clin Psychol 53:88–94. 10.1037/0022-006X.53.1.88 3980834 10.1037//0022-006x.53.1.88

[56] Sarwer DB, Wadden TA, Whitaker LA (2002) An investigation of changes in body image following cosmetic surgery. Plast Reconstr Surg 109(1):363–369. 10.1097/00006534-200201000-00060 11786842 10.1097/00006534-200201000-00060

[57] Swami V, Kannan K, Furnham A (2011) Positive body image: inter-ethnic and rural-urban differences among an indigenous sample from Malaysian Borneo. Int J Soc Psychiatry 58:568–576 21821633 10.1177/0020764011415208

[58] Claes L, Hart TA, Smits D et al (2012) Validation of the social appearance anxiety scale in female eating disorder patients. Eur Eat Disord Rev 20:406–409. 10.1002/erv.1147 21805536 10.1002/erv.1147

[59] Cuzzolaro M, Vetrone G, Marano G, Garfinkel PE (2006) The body uneasiness test (BUT): development and validation of a new body image assessment scale. Eat Weight Disord 11:1–13. 10.1007/BF03327738 16801740 10.1007/BF03327738

[60] Muehlenkamp JJ, Peat CM, Claes L, Smits D (2012) Self-injury and disordered eating: expressing emotion dysregulation through the body. Suicide Life Threat Behav 42:416–425. 10.1111/j.1943-278X.2012.00100.x 22646483 10.1111/j.1943-278X.2012.00100.x

[61] Veale D (2001) Cognitive–behavioural therapy for body dysmorphic disorder. Adv Psychiatr Treat 7:125–132. 10.1192/apt.7.2.125

[62] Pope HG, Phillips KA, Olivardia R (2002) The Adonis complex: how to identify, treat and prevent body obsession in men and boys. Simon & Schuster, New York

[63] Gervais SJ, Bernard P, Klein O, Allen J (2013) Toward a unified theory of objectification and dehumanization. Nebr Symp Motiv 60:1–23. 10.1007/978-1-4614-6959-9_1 23947276 10.1007/978-1-4614-6959-9_1

[64] LaCroix JM, Pratto F (2015) Instrumentality and the denial of personhood: the social psychology of objectifying others. Revue inter de psychologie sociale 28:183–211

[65] Price TF, Peterson CK, Harmon-Jones E (2012) The emotive neuroscience of embodiment. Motiv Emot 36:27–37. 10.1007/s11031-011-9258-1

[66] World Medical Association (2013) World Medical Association Declaration of Helsinki: ethical principles for medical research involving human subjects. JAMA 310:2191–2194. 10.1001/jama.2013.281053 24141714 10.1001/jama.2013.281053

[67] Cuzzolaro M, Vetrone G, Merano G, Battacchi MW (1999) BUT: una nuova scala per la valutazione del disagio relativo all’immagine del corpo. Psichiatria dell’infanzia e dell’adolescenza 417–428

[68] Cella S, Cipriano A, Aprea C, Cotrufo P (2021) Self-esteem and binge eating among adolescent boys and girls: the role of body disinvestment. Int J Environ Res Public Health 18:7496. 10.3390/ijerph18147496 34299947 10.3390/ijerph18147496 PMC8304970

[69] Cipriano A, Cella S, Cotrufo P (2020) Non-suicidal self-injury among Italian adolescents: the role of parental rejection, self-concept, anger expression, and body investment. Clin Neuropsychiatry 17:330–338. 10.36131/cnfioritieditore20200602 34909011 10.36131/cnfioritieditore20200602 PMC8629071

[70] McKinley NM, Hyde JS (1996) The objectified body consciousness scale: development and validation. Psychol Women Q 20:181–215. 10.1111/j.1471-6402.1996.tb00467.x

[71] Dakanalis A, Timko AC, Clerici M et al (2017) Objectified body consciousness (OBC) in eating psychopathology. Assessment 24:252–274. 10.1177/1073191115602553 26336907 10.1177/1073191115602553

[72] Berger VW, Zhou Y (2014) Kolmogorov-Smirnov test: overview. In: Wiley StatsRef: Statistics Reference Online. John Wiley & Sons Ltd, Hoboken

[73] Kateri M (2014) Contingency table analysis: methods and implementation using R. Springer, New York

[74] Rutherford A (2011) ANOVA and ANCOVA: A GLM approach. John Wiley & Sons, Hoboken

[75] Quade D (1967) Rank analysis of covariance. J Am Stat Assoc 62:1187–1200. 10.1080/01621459.1967.10500925

[76] Rheinheimer DC, Penfield DA (2001) The effects of type I error rate and power of the ANCOVA f test and selected alternatives under nonnormality and variance heterogeneity. J Exp Educ 69:373–391. 10.1080/00220970109599493

[77] Hayes AF (2017) Introduction to mediation, moderation, and conditional process analysis, second edition: a regression-based approach. Guilford Publications, New York

[78] Fredrickson BL, Roberts T-A (1997) Objectification theory: toward understanding women’s lived experiences and mental health risks. Psychol Women Q 21:173–206. 10.1111/j.1471-6402.1997.tb00108.x

[79] Mercurio AE, Landry LJ (2008) Self-objectification and well-being: the impact of self-objectification on women’s overall sense of self-worth and life satisfaction. Sex Roles 58:458–466. 10.1007/s11199-007-9357-3

[80] Bromberg PM (2003) Something wicked this way comes: Trauma, dissociation, and conflict: the space where psychoanalysis, cognitive science, and neuroscience overlap. Psychoanal Psychol 20:558–574. 10.1037/0736-9735.20.3.558

[81] Joyce RA (2023) Body modification: rituals, gender, and symbolism. In: Manni F, d’Errico F (eds) The oxford handbook of the archaeology and anthropology of body modification. Oxford University Press, Oxford

[82] Martin-Seaver M (2019) First-personal body aesthetics as affirmations of subjectivity. Contemp Aesthet (J Arch) 17:12

[83] Mucci C, Scalabrini A (2021) Traumatic effects beyond diagnosis: the impact of dissociation on the mind–body–brain system. Psychoanal Psychol 38:279–289. 10.1037/pap0000332

[84] von Soest T, Kvalem IL, Roald HE, Skolleborg KC (2009) The effects of cosmetic surgery on body image, self-esteem, and psychological problems. J Plast Reconstr Aesthet Surg 62:1238–1244. 10.1016/j.bjps.2007.12.093 18595791 10.1016/j.bjps.2007.12.093

[85] Namir S (2006) Embodiments and disembodiments: the relation of body modifications to two psychoanalytic treatments. Psychoanal Cult Soc 11:217–223. 10.1057/palgrave.pcs.2100085

[86] Preece C, Rodner V, Whittaker L (2023) Multiple embodiment relations: sense-making in dissociative experiences. Consum Mark Cult. 10.1080/10253866.2023.2221635

[87] Swami V (2011) Marked for life? A prospective study of tattoos on appearance anxiety and dissatisfaction, perceptions of uniqueness, and self-esteem. Body Image 8:237–244. 10.1016/j.bodyim.2011.04.005 21641893 10.1016/j.bodyim.2011.04.005

[88] Favazza AR (1996) Bodies under siege: self-mutilation and body modification in culture and psychiatry. JHU Press, Baltimore

[89] Le Breton D (2018) Understanding skin-cutting in adolescence: sacrificing a part to save the whole. Body Soc 24:33–54. 10.1177/1357034X18760175

[90] Menzel JE, Sperry SL, Small B et al (2011) Internalization of appearance ideals and cosmetic surgery attitudes: a test of the tripartite influence model of body image. Sex Roles 65:469–477. 10.1007/s11199-011-9983-7

[91] Laricchiuta D, Garofalo C, Mazzeschi C (2023) Trauma-related disorders and the bodily self: current perspectives and future directions. Front Psychol. 10.3389/fpsyg.2023.1166127 37275691 10.3389/fpsyg.2023.1166127 PMC10235635

[92] Sierra M, David AS (2011) Depersonalization: a selective impairment of self-awareness. Conscious Cogn 20:99–108. 10.1016/j.concog.2010.10.018 21087873 10.1016/j.concog.2010.10.018

[93] Maxwell SE, Cole DA (2007) Bias in cross-sectional analyses of longitudinal mediation. Psychol Methods 12:23–44. 10.1037/1082-989X.12.1.23 17402810 10.1037/1082-989X.12.1.23

[94] Podsakoff PM, MacKenzie SB, Lee J-Y, Podsakoff NP (2003) Common method biases in behavioral research: a critical review of the literature and recommended remedies. J Appl Psychol 88:879–903. 10.1037/0021-9010.88.5.879 14516251 10.1037/0021-9010.88.5.879

[95] Bethlehem J (2010) Selection bias in web surveys. Int Stat Rev 78:161–188. 10.1111/j.1751-5823.2010.00112.x

[96] Briere J, Weathers FW, Runtz M (2005) Is dissociation a multidimensional construct? Data from the multiscale dissociation inventory. J Trauma Stress 18:221–231. 10.1002/jts.20024 16281216 10.1002/jts.20024

